# Multimodal Information Fusion for Control of Rehabilitation Robots in Motor Dysfunction: A Review

**DOI:** 10.3390/bioengineering13060627

**Published:** 2026-05-27

**Authors:** Chang Liu, Xiaoyan Wang, Mostafa Orban, Alexander Vartanov, Mahmoud Elsamanty, Tingrui Pan, Kai Guo

**Affiliations:** 1 School of Biomedical Engineering (Suzhou), University of Science and Technology of China, Hefei 230022, China; 2 Suzhou Institute of Biomedical Engineering and Technology, Chinese Academy of Sciences, Suzhou 215163, China; mustafa.essam@feng.bu.edu.eg; 3 Faculty of Psychology, Lomonosov Moscow State University, Moscow 125009, Russia; wangxiaoyan99@mail.ru (X.W.);; 4 Mechanical Department, Faculty of Engineering at Shoubra, Benha University, Cairo 11629, Egypt; 5 Suzhou Institute for Advanced Research, University of Science and Technology of China, Suzhou 215123, China; 6 Tsinghua Shenzhen International Graduate School, Tsinghua University, Shenzhen 518055, China; 7 Chongqing Guoke Medical Innovation Technology Development Co., Ltd., Chongqing 400799, China

**Keywords:** information fusion, rehabilitation robotics, motor dysfunction, data-level fusion, feature-level fusion, decision-level fusion

## Abstract

This paper reviews recent advances in assistive devices based on multimodal information fusion control, designed for individuals with motor dysfunction. The prevalence of motor dysfunction is increasingly concerning amidst global population aging. Information fusion technology, widely adopted in rehabilitation, enhances the efficacy and specificity of rehabilitation treatments. This paper introduces the concept of multimodal information fusion control into rehabilitation equipment design. It highlights the advantages and disadvantages of data-level, feature-level, and decision-level fusion, along with commonly employed fusion algorithms. By summarizing and analyzing the current state of research, this paper aims to provide a valuable reference for the further development and optimization of assistive devices for motor dysfunction.

## 1. Introduction

Motor dysfunction—broadly defined as any impairment that limits the planning, execution, or coordination of voluntary movement—is a leading cause of long-term disability worldwide and represents a growing public health concern as the global population ages [1,2]. Its etiologies span a wide spectrum, including stroke, spinal cord injury, traumatic brain injury, and neurodegenerative diseases such as Parkinson’s disease and multiple sclerosis [3,4]. Among these, stroke is the single most prevalent cause. Research projections indicate that, globally, the numbers of ischemic stroke cases, deaths, and DALYs (disability-adjusted life years) increased from 4.07, 2.05, and 40.50 million, respectively, in 1990 to 7.86, 3.15, and 62.53 million, respectively, in 2020, and are projected to reach 9.62 million cases by 2030 [5,6].

Seventy-five percent of stroke survivors are left with varying degrees of motor dysfunction [5,6]. More broadly, regardless of etiology, limb motor dysfunction is mostly attributable to localized neurological damage in the brainstem or to injury of the central nervous system [1,7]. Experimental results show that the human central nervous system has a high degree of plasticity, and specific functional training can promote central nervous reorganization and compensation, thus restoring the limb motor function of patients [1,8,9].

Addressing motor function impairments is of paramount importance in improving the quality of daily activities for patients during the later stages of their recovery [10]. By designing assistive devices for individuals with motor dysfunction arising from diverse neurological conditions, real-time movement assistance and rehabilitation training can be provided to enhance their motor abilities, balance, and coordination [11]. This not only improves the patients’ quality of life, but also reduces healthcare costs and societal burden [2,12].

A comprehensive and effective rehabilitation robot system should include precise mechanical structures capable of accurately implementing limb traction trajectory planning, a control system with various rehabilitation training modes, and a perception system capable of accurately interpreting patient movements [13,14].

According to modern rehabilitation medicine theory, the reconstruction of limb motor function in patients is primarily based on the principles of neural plasticity, which involves stimulating the reorganization and compensation of the central nervous system [4,15]. Therefore, assisting patients in actively participating in rehabilitation training by identifying their movement intentions is highly beneficial for the reconstruction of their nervous system and the recovery of motor function [16].

For these reasons, research on rehabilitation robot perception systems and intelligent assistive systems has been increasing in recent years. The main objective is to perceive the patients’ movements, limb force/position, electroencephalogram (EEG), electromyogram (EMG), and other information to understand their movement intentions and enhance rehabilitation training [17,18,19,20,21,22].

The perception system of a rehabilitation robot needs to acquire various aspects of the current status, including the movements, physiological parameters, and interactive forces between the patient and the robot, as well as accurately understand the patient’s movement intentions to achieve interactive control between the patient and the robot [23,24,25].

Additionally, it must continuously monitor for any abnormal patient states to ensure human–robot safety.

For a rehabilitation robot system, the perception system is not only a crucial component of the human–robot closed-loop control, but also the source of input signals for the robot’s compliant control. Furthermore, it serves as the primary basis for comprehensive performance evaluation of the rehabilitation robot, making it essential when addressing topics such as spasm detection, exercise-induced muscle fatigue, and motion classification [26].

Although numerous review articles have been published on the application of multimodal information fusion technology in rehabilitation robots, few studies systematically detail its specific technical classifications. This includes different fusion levels (data level, feature level, decision level), system architectures, commonly used algorithms, and a comprehensive analysis of recent rehabilitation devices.

This paper systematically reviews the current state of multimodal information fusion control technology in rehabilitation devices for motor dysfunction, outlining its key technical components. These include the three levels of information fusion, diverse system architectures, and multiple representative algorithms.

The remainder of this paper is organized as follows: Section 2 describes the literature search methodology. Section 3 explains the rehabilitation mechanisms for motor dysfunction, particularly the Brunnstrom stages of recovery. Section 4 details the core technologies of multimodal information fusion, including its fusion levels, system architectures, and commonly used algorithms. Section 5 introduces specific applications and representative devices of this technology in recent rehabilitation equipment. Section 6 analyzes the current research trends, challenges, and technical trade-offs. Finally, Section 7 summarizes the entire paper and suggests future research directions.

## 2. Methods

**Figure 1:**
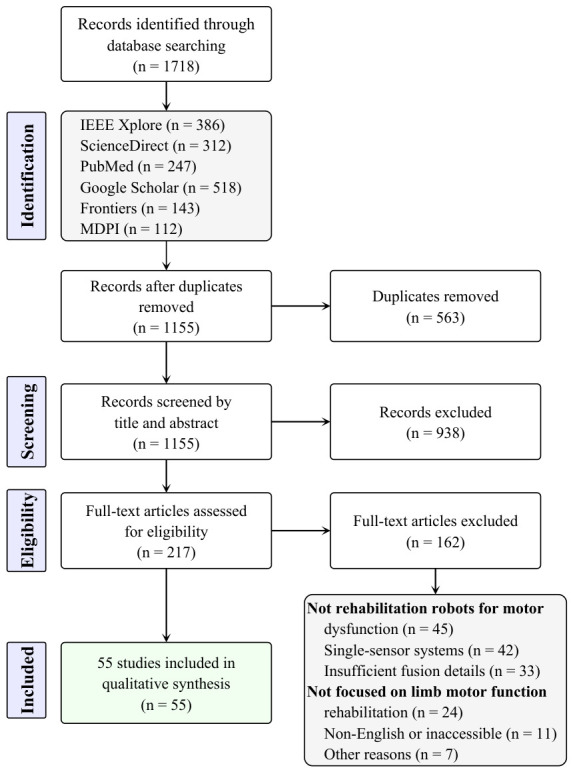
PRISMA flow diagram illustrating the literature selection process.

This review was conducted with reference to the Preferred Reporting Items for Systematic Reviews and Meta-Analyses (PRISMA) framework to improve the transparency and reproducibility of the literature selection process. The aim of the search was to identify studies on multimodal information fusion for the control, assistance, or assessment of rehabilitation robotic systems in individuals with motor dysfunction. The complete literature selection process is summarized in the PRISMA flow diagram shown in Figure 1.

### 2.1. Search Strategy

A systematic literature search was performed across six electronic databases: IEEE Xplore, ScienceDirect, PubMed, Google Scholar, Frontiers, and MDPI. The search covered publications from January 2010 to December 2024. Because the scope of this review is motor dysfunction in general rather than a single disease category, the search strategy combined general terms related to motor dysfunction with representative etiology-related terms. The following Boolean search expression was applied to titles, abstracts, and keywords where supported by the database:

(“motor dysfunction” OR “motor impairment” OR “motor deficit” OR “movement disorder” OR “limb dysfunction” OR “stroke” OR “post-stroke” OR “spinal cord injury” OR “traumatic brain injury” OR “cerebral palsy” OR “Parkinson’s disease” OR “multiple sclerosis”) AND (“rehabilitation robot” OR “rehabilitation robotics” OR “rehabilitation exoskeleton” OR “assistive robot” OR “assistive device” OR “wearable robot”) AND (“information fusion” OR “data fusion” OR “sensor fusion” OR “multimodal” OR “multi-sensor integration”).

Disease-specific terms, such as “stroke,” “spinal cord injury,” and “Parkinson’s disease,” were included to improve the sensitivity of the search and to avoid missing relevant rehabilitation robotic studies that described the clinical population by etiology rather than by the broader term “motor dysfunction.” These terms were not used as eligibility requirements. Accordingly, studies were not required to involve stroke patients; rather, eligible studies had to address motor dysfunction rehabilitation, assistance, or functional assessment using multimodal information fusion.

In addition to the database search, the reference lists of relevant review articles and highly cited primary studies were manually screened to identify additional eligible publications. When multiple publications described substantially overlapping systems or datasets, the most complete and technically detailed version was retained for analysis.

### 2.2. Study Selection Process

The initial database search yielded a total of 1718 records, including 386 from IEEE Xplore, 312 from ScienceDirect, 247 from PubMed, 518 from Google Scholar, 143 from Frontiers, and 112 from MDPI. After removing 563 duplicate records using reference management software and manual verification, 1155 unique records remained for title and abstract screening.

In the first screening stage, the titles and abstracts of these 1155 records were reviewed according to the inclusion and exclusion criteria described below. Records that were clearly unrelated to rehabilitation robotics, multimodal information fusion, or motor function rehabilitation were excluded. This stage resulted in the exclusion of 938 records. The remaining 217 articles were retrieved in full text for detailed eligibility assessment.

In the second stage, the full texts of the 217 articles were evaluated against the complete eligibility criteria. Of these, 162 articles were excluded for the following reasons:

- Not specifically addressing rehabilitation robots or assistive robotic devices for motor dysfunction (n=45);
- Single-sensor systems without multimodal information fusion (n=42);
- Insufficient technical details on fusion methods or sensor integration (n=33);
- Not focused on limb motor function rehabilitation, assistance, or assessment (n=24);
- Non-English publications or full text inaccessible to the authors (n=11);
- Other reasons, including editorials, duplicated conference/journal content, or conference abstracts without sufficient technical information (n=7).

After full-text assessment, 55 studies met all eligibility criteria and were included in the qualitative synthesis. These studies form the basis of the detailed summary and analysis in Section 5.

### 2.3. Inclusion Criteria

The inclusion criteria were as follows:

- The study focused on rehabilitation robots, wearable robotic systems, robotic orthoses, prosthetic control systems, or assistive robotic devices intended for individuals with motor dysfunction.
- The study involved multimodal information fusion, defined as the integration of two or more distinct signal sources or sensor modalities, such as surface electromyography (sEMG), EEG, inertial measurement units (IMUs), force sensors, pressure sensors, joint encoders, vision, near-infrared spectroscopy (NIRS), electrocardiography (ECG), or related physiological and biomechanical signals.
- The study provided sufficient technical information on the multimodal sensing architecture, fusion level, fusion algorithm, classification method, control strategy, or assessment framework.
- The target application was related to limb motor rehabilitation, motor assistance, movement intention recognition, prosthetic/exoskeleton control, gait analysis, hand function rehabilitation, or quantitative functional assessment.
- Articles were English-language peer-reviewed journal papers or conference papers accessible to the authors.

### 2.4. Exclusion Criteria

The exclusion criteria were as follows:

- Studies that did not address rehabilitation robots, assistive robotic systems, robotic orthoses, prosthetic control, or wearable robotic devices for motor dysfunction.
- Studies based on a single sensor modality only, without multimodal information fusion.
- Studies lacking sufficient information on sensor integration, fusion strategy, or control/assessment algorithms.
- Studies focused on non-motor rehabilitation domains, such as cognitive rehabilitation, speech therapy, or purely diagnostic imaging without robotic control or motor function assessment.
- Studies involving general human activity recognition, sports monitoring, or healthy-subject motion analysis without clear relevance to motor dysfunction rehabilitation or assistive control.
- Review articles, editorials, opinion papers, patents, and abstracts without full technical content. These documents were used only for background understanding when appropriate and were not included in the primary analysis.
- Non-English publications or articles whose full texts were inaccessible.

### 2.5. Analytical Framework

An analytical framework was developed to organize the 55 included studies. Each study was categorized according to four dimensions: (1) the multimodal signal combination employed, such as EEG–EMG, sEMG–IMU, sEMG–force/angle sensors, kinematic–mechanical sensor fusion, or vision-augmented fusion; (2) the level of information fusion, including data-level, feature-level, or decision-level fusion; (3) the algorithmic approach, such as traditional machine learning, fuzzy logic, Bayesian fusion, neural networks, convolutional neural networks (CNNs), long short-term memory (LSTM) networks, attention-based models, or adaptive control; and (4) the target application, including upper limb rehabilitation, hand function rehabilitation, lower limb exoskeleton control, prosthetic control, gait analysis, or quantitative functional assessment.

This analytical framework provides the basis for the device-level summary in Section 5 and the signal-combination-based discussion therein. By organizing the literature in this way, this review not only aims to synthesize individual device implementations, but also broader technical trends in multimodal fusion control for motor dysfunction rehabilitation.

## 3. Rehabilitation Mechanisms for Motor Dysfunction

Motor dysfunction can arise from a variety of neurological conditions, including stroke, spinal cord injury, traumatic brain injury, and neurodegenerative diseases. Despite their differing pathophysiologies, these conditions share a common consequence: disruption of descending motor pathways that results in clinical manifestations such as altered muscle tone, decreased muscle strength, impaired coordination, and loss of balance, all of which severely affect patients’ ability to perform activities of daily living [1,3,27]. The severity of motor dysfunction varies across individuals; mild cases may present only as motor abnormalities, while severe cases may lead to paralysis or complete incapacitation [28,29,30,31].

A fundamental principle underlying rehabilitation for motor dysfunction, regardless of etiology, is neural plasticity: the capacity of the central nervous system to reorganize its structure and function in response to targeted, repetitive functional training [1,4,15]. Rehabilitation programs therefore need to be tailored to each patient’s specific impairment profile to facilitate neural reorganization and help them regain motor function and improve their quality of life [32].

**Figure 2:**
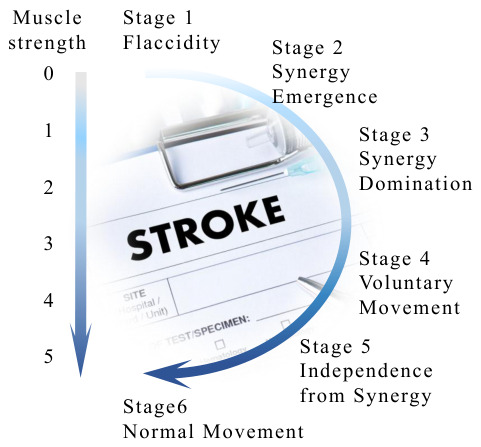
Brunnstrom rehabilitation stages.

Because stroke is the most prevalent cause of acquired motor dysfunction, its recovery process has been the most extensively characterized. The Brunnstrom stages of rehabilitation, developed by Signe Brunnstrom in the 1960s, provide a widely adopted clinical framework that describes the progressive recovery of motor function after stroke in six stages, each representing a distinct motor pattern and functional performance level [33,34,35]. Although originally designed for stroke, the staged perspective of progressing from flaccidity through synergy dominance to voluntary control has also informed rehabilitation goal-setting for other neurological conditions. The six stages are briefly described as follows (Figure 2):

Stage 1 (Flaccidity): At this stage, the patient exhibits impaired reflex activity, muscle laxity, and no active motor ability.

Stage 2 (Synergy Emergence): The patient begins to exhibit basic synergy patterns, i.e., uncoordinated but sequential activity of the limb muscle groups. These patterns are known as motor merging patterns or “muscle synergies.” They are usually planned, repeated movements.

Stage 3 (Synergy Domination): At this stage, motor merging patterns become clearer and start to affect more muscle groups. The patient can do some coordinated movements in the synergistic pattern, but only for a short time.

Stage 4 (Voluntary Movement): At this stage, the patient is able to gradually depart from the merged movement pattern and begin making some voluntary, non-merged movements. These movements are no longer totally limited to the synergistic pattern, though they may still be constrained.

Stage 5 (Independence from Synergy): The patient is now able to depart from the combined movement pattern and begins to move their joints more independently. Movement accuracy and coordination improve with time.

Stage 6 (Normal Movement): At this stage, the patient’s motor function has largely recovered to a level comparable to that of a healthy individual. The ability to control, coordinate, and move is similar to that of healthy people.

It is important to note that the Brunnstrom stages do not prescribe a universal recovery trajectory; each patient’s recovery is different and depends on numerous individual factors, including etiology, lesion location, age, and comorbidities. Nonetheless, the classification system can help the rehabilitation team evaluate a patient’s motor recovery progress and formulate a personalized rehabilitation plan [36].

## 4. Multimodal Information Fusion Technologies

Information fusion is the use of computer technology to carry out multi-level, intelligent, and comprehensive processing of information from different sensors to obtain more comprehensive and accurate decision-making results than relying on a single source [37,38]. Therefore, information fusion takes the data collected by sensors as the data source and the data information as the processing object, and its core is the coordinated optimization and comprehensive processing of multimodal information [39].

**Figure 3:**
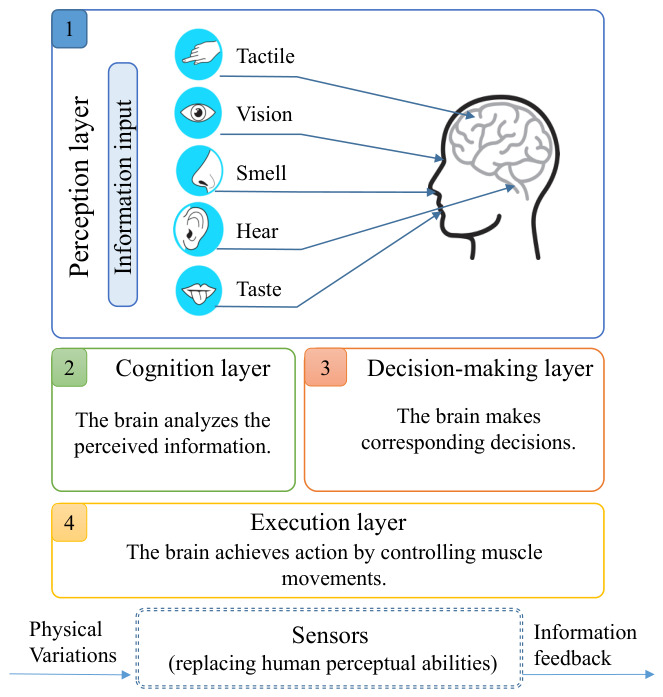
Principles of multimodal information fusion technology.

For example, EEG–EMG fusion is to integrate and process the information from the brain and muscles according to certain rules, remove redundancy, and make full use of their coordinated complementarity to improve the reliability and accuracy of motor pattern recognition [40]. The general principle of multimodal information fusion is illustrated in Figure 3. The process of information fusion can be categorized into three levels according to the level of abstraction of the source data: data-level fusion, feature-level fusion, and decision-level fusion [41].

Data-level fusion: directly fuses the raw data collected from multiple sensors and then performs feature extraction and pattern recognition on the fused data [42]. As the lowest level of fusion, it preserves the maximum amount of original information with higher fineness [43], but at the cost of high computational complexity, poor real-time performance, and limited anti-interference ability.

In practice, fusing heterogeneous sensor data requires three essential preprocessing steps. First, since different sensors typically operate at different sampling rates and physical units—for example, sEMG at 1000 Hz in millivolts versus IMU at 200 Hz in m/s^2^—the raw data streams must be temporally synchronized through resampling or interpolation to a common time base. Second, the signals are normalized (e.g., z-score or min–max scaling) to eliminate scale discrepancies across modalities. Third, the aligned and normalized streams are concatenated, either by channel-wise stacking to form a multi-channel input matrix or by appending them into a single augmented vector at each time step.

The resulting unified representation is then passed to downstream feature extraction and classification stages. For instance, synchronized sEMG and IMU recordings during walking can be stacked into a multi-channel time-series matrix and processed by a CNN that jointly extracts spatial and temporal patterns from both modalities [44,45]. Although this approach allows for the model to discover cross-modal correlations directly from raw data, it imposes strict requirements on synchronization accuracy and substantially increases input dimensionality. Commonly used data-level fusion algorithms include weighted averaging, fuzzy logic [46], and wavelet transform [47].

**Figure 4:**
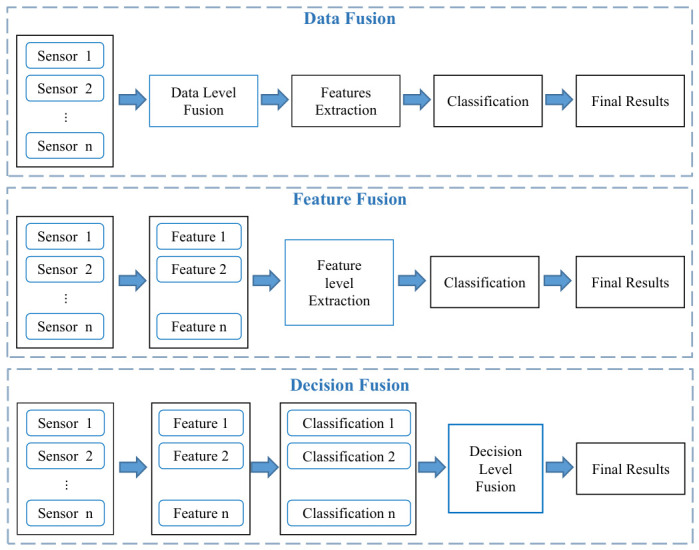
Classification of information fusion.

Feature-level fusion: extracts the features of the data collected by multiple sensors separately, and then the feature vectors are fused with certain rules of association before making judgment decisions [48]. This fusion method refines and compresses the original sensor information, makes the patterns within the observed data more apparent, and at the same time improves the computing speed. However, the design and selection of effective features often require domain expertise, and information loss may occur during the feature extraction stage. Commonly used feature-level information fusion algorithms include principal component analysis (PCA), independent component analysis (ICA), autoencoder, feature fusion neural network, etc. [49]. As illustrated in Figure 4, after the feature vectors from individual modalities are concatenated or combined, a feature-level extraction step is typically performed before classification. This step applies dimensionality reduction or feature transformation techniques—such as PCA, ICA, or autoencoder-based methods—to the fused high-dimensional feature vector. Its purpose is to remove redundant or correlated components introduced by the multi-source concatenation and to extract a compact, discriminative feature representation that improves the subsequent classifier’s performance and computational efficiency.

Decision-level fusion: infers the final decision by fusing the sub-decision results after feature extraction and classification of the data collected by multiple sensors respectively [50]. The advantages of this method are that the sensor results can be selected flexibly, which improves the fault-tolerance of the system; the ability to accommodate multiple sources of heterogeneous sensors is enhanced; the amount of fused information calculation is reduced, and the real-time performance of the system is improved [51]. However, since the fusion center only receives locally processed decision results rather than raw data, detailed inter-modal correlations may be lost. Commonly used decision-level information fusion algorithms include Dempster–Shafer (D-S) evidence theory, Bayesian decision theory, weighted fusion, voting fusion, etc. [49,52]. A schematic comparison of these three fusion levels is presented in Figure 4.

**Figure 5:**
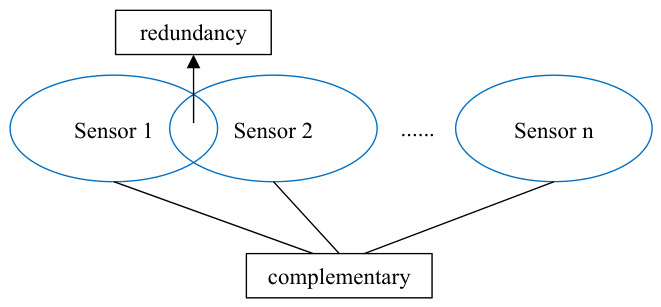
Sensor information classification.

As shown in Figure 5, in general, the types of multimodal information can be categorized as follows, depending on the information provided by each sensor:

Redundant information: By having multiple sensors of the same or different types together providing information about some aspect of the environment or the robot’s own state, multiple copies of that information can be obtained, and by fusing these copies of the data, the effects of various types of noise can be attenuated. The use of redundant information also improves the reliability of the system, as redundant information allows for the robot to utilize the information provided by other sensors to make inference decisions even if a sensor has a problem while the robot is operating, thus avoiding the phenomenon of a single-sensor system where the robot is paralyzed in the event of a failure of that sensor.

Complementary information: By a number of the same or different types of sensors respectively providing different aspects of the environment or the robot’s own state information, they are relatively independent of each other, but together can constitute a more complete description of the environment or the robot’s own state. Complementary information can make up for the limitation that a single sensor can only provide information on a certain aspect so that the robot can obtain a more comprehensive knowledge of the environment or its own state, thus improving the robot’s ability to make correct decisions.

### 4.1. Common Control Signals for Rehabilitation Equipment

Rehabilitation robots are a type of robot system that involves close human–machine interaction and coupling. Therefore, identifying the wearer’s movement intention is a prerequisite for controlling rehabilitation robots. At present, the interaction between humans and rehabilitation robots can be divided into methods based on motion sensors, methods based on motion images, and methods based on bioelectrical signals, according to the way the wearer’s intended movements are performed.

The first method based on sensors is to obtain joint inertial information, pressure information, and other information through sensors, and to identify the wearer’s movement intention by processing the data. However, the information can only be collected after the wearer has exercised, which introduces inherent latency.

The second method based on motion images is to obtain motion information through the processing of motion images, but this method has limitations in the recognition scene.

The third method based on bioelectrical signals mainly uses bioelectrical signals such as electroencephalogram signals and surface electromyogram signals to realize the recognition of the wearer’s intention.

**Figure 6:**
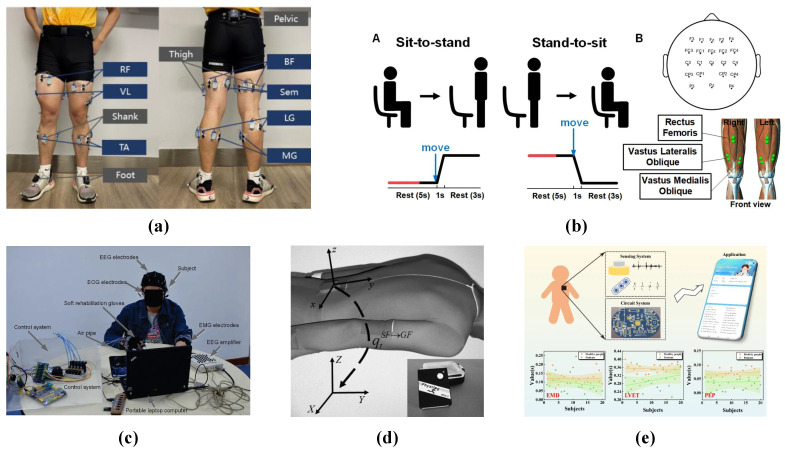
Representative acquisition setups for common control signals in rehabilitation systems. (**a**) sEMG and IMU sensor placement on the lower limb for locomotion mode recognition. Adapted with permission from Ref. [54]. Copyright 2021, the authors; (**b**) EEG and EMG signal acquisition paradigm for detecting pre-movement intention of sitting and standing. Adapted with permission from Ref. [55]. Copyright 2025, Li, Xu, Feng, Wang, Zhang, Zhang, Cheng, Chen, Chen and Zhang; (**c**) EEG/EMG/EOG-based multimodal human–machine interface electrode placement and signal acquisition for real-time soft robot hand control. Adapted with permission from Ref. [56]. Copyright 2019, Zhang, Wang, Zhang, Xiao and Wang; (**d**) IMU sensor placement on the lower limb for inertial motion measurement with coordinate frame definition. Adapted with permission from Ref. [57]. Copyright 2012, the authors; (**e**) Wearable device integrating ECG and PCG sensors for cardiac health monitoring. Adapted with permission from Ref. [58]. Copyright 2025, The Author(s).

The generation of bioelectrical signals precedes actual limb movement, which effectively solves the problem of signal acquisition lag. However, human bioelectrical signals have certain complexity and uncertainty, and the accuracy and reliability of using bioelectrical signals alone as the exoskeleton perception and control signals are low. Therefore, the rehabilitation robot control scheme that integrates multi-source signals, taking into account bioelectrical and kinematic information, can achieve superior speed and accuracy. Therefore, this section will introduce several control signals commonly used in rehabilitation robot control schemes [53]. Representative signal acquisition setups and processing pipelines for each control signal modality are illustrated in Figure 6.

#### 4.1.1. Electromyography (EMG)

Electromyography (EMG) is a kind of bioelectric signal generated in the process of human muscle activity, which is the comprehensive result of the conduction and superposition of different motor unit action potentials (MUAP) in muscle fibers and cell tissues [59]. A representative sEMG and IMU sensor placement for multimodal locomotion recognition is shown in Figure 6a. Skeletal muscle is the most abundant muscle tissue in the human body, accounting for about 40% of the human body weight. It is usually attached to the bone through the tendon, and is so named because of this attachment to bone. Because the contraction of skeletal muscle is controlled by human will, almost all the movements that human beings can complete are related to skeletal muscle, which is also the muscle type commonly studied in the field of rehabilitation robots.

The detection method of EMG signal is divided into two types according to the type of guiding electrode and the detection and placement form. One is to use needle electrodes as the guiding electrodes and detect the electrical activity directly near the active muscle fibers by inserting them into the muscle tissue. The EMG signal obtained by this detection method is called needle EMG signal (NEMG) [60].

The other is to indirectly measure the potential changes caused by muscle activity by closely contacting the skin surface in the area where the active muscle is located through the surface electrode. The EMG signal obtained by this detection method is called surface EMG signal (sEMG). Because the cross-section of the needle electrode is very small, the sampling is inserted into the muscle to directly contact the muscle fiber, so what is recorded is the potential activity of a small number of muscle fibers near the surface of the detection electrode. Compared with surface electrodes, needle electrode EMG signals have a better spatial resolution and a higher signal-to-noise ratio. The amplitude of the NEMG signal typically ranges from 0 to 10 mV (peak-to-peak). Its bandwidth spans from 2 Hz to 10 kHz, with the predominant energy distribution concentrated within the 20 Hz–4 kHz range. Furthermore, the MUAP process time typically lasts between 3 and 6 ms.

The invasive detection method of needle electrodes will cause certain damage to muscle and fat tissues. The measurement generally requires the participation of doctors or professional nursing staff, and causes pain to the measured object. It is not easy to measure multiple channels, repeatedly or for a long time, and it is not suitable for use as a human–computer interaction means in daily life.

Compared with needle electrodes, surface electrodes have a larger detection surface and lower spatial resolution. The signal recorded by the surface electrode is the comprehensive effect of the electrical activity of muscle fibers within a certain range. The amplitude range of sEMG signal is 0–1.5 mV, the bandwidth is 0.5–2 kHz, the energy is mainly distributed between 50–200 Hz, and the process time of MUAP is generally between 5–20 ms. The greatest advantage of surface electrode measurement of EMG signals is the non-invasive measurement method without damage. The operation is simple and easy, and no professional medical personnel are required to participate. This has made the research and application based on surface EMG signals the mainstream in the field of EMG signals [61,62].

#### 4.1.2. Electroencephalography (EEG)

Brain–computer interface (BCI) has attracted wide attention in the field of human–computer interaction in recent years [63]. It can directly decode the user’s EEG signal to control the device, and has become a new control method. However, unlike sEMG signals, which have a mapping relationship with movement intention or limb movement, EEG signals do not fully reflect this explicit direct relationship. Therefore, extracting motor intention from EEG signals is more challenging than extracting motor intention from surface EMG signals [64,65]. A representative EEG and EMG signal acquisition paradigm for detecting pre-movement intention is illustrated in Figure 6b. There are many ways to collect EEG signals, and they can be classified according to various criteria. The common acquisition methods include implantable and non-implantable methods. As the name suggests, implanted acquisition aims to implant the signal acquisition device into the cerebral cortex so that the quality of the acquired EEG signal is high and the positioning of the brain area is more specific. However, invasive brain–computer interfaces have the risk of surgical complications and infection, and long-term signal instability and performance degradation in decoding intention information. At present, they are mainly used for animal experiments.

In contrast, non-invasive methods do not require the implantation of electrodes inside the brain, making them safer and more convenient. Non-invasive methods collect data through metal lead electrodes on the EEG cap. When neurons in the brain of a person or a creature are active, they will produce charged ions. When the charged ions are active near the lead electrodes on the cerebral cortex, they will cause a potential difference between the lead metal electrodes, thus generating electrical signals. Because the generated electrical signal is unstable, in order to improve the stability of the signal, it is necessary to remove the stratum corneum of the skin under the lead electrode. At the same time, the use of electrolyte gel (EEG paste) can form a good electrical contact between the metal electrode and the skin [66].

The intensity of EEG signals is relatively small, the frequency is roughly distributed between 0.5 and 50 Hz, the amplitude is usually about 50 μV, and the maximum does not exceed 200 μV. According to the frequency range, EEG signals can be roughly divided into five bands, namely δ (0.5–4 Hz), θ (4–8 Hz), α (8–13 Hz), β (13–30 Hz), and γ (>30 Hz). Among them, the α and β bands are closely related to motor imagery and execution, while the γ band has also been shown to play an important role in motor-related BCI research. Different emotional states of normal people will affect the frequency of EEG signals generated by the brain. Because EEG signals are easily affected by other internal bioelectric signals such as electrooculogram signals and electrocardiogram signals, EEG signals are highly random. At the same time, due to the changes in human physiological factors, the frequency and amplitude of EEG signals at different times are different, and they have the characteristics of non-stationary signals, which makes it difficult to collect EEG signals.

#### 4.1.3. Electrooculography (EOG)

Electrooculography (EOG) measures the potential difference between the cornea (positive) and the retina (negative), which ranges from 0.4 to 1.0 mV and varies with the direction of eyeball rotation [67]. Horizontal EOG is recorded by electrodes placed approximately 2.5 cm from the outer canthus, while vertical EOG is captured by electrodes above the eyebrow and below the lower eyelid. The signal amplitude changes approximately linearly with the sine of the rotation angle for angles below 30°, with linearity degrading beyond this range [68]. A representative multimodal EEG/EMG/EOG electrode placement and signal acquisition setup is shown in Figure 6c.

It should be noted that, unlike EMG and EEG, EOG is rarely used as a direct control signal for limb motor rehabilitation robots. Its primary role in assistive technology has been in communication interfaces and environmental control systems for individuals with severe motor impairments (e.g., high-level spinal cord injury or locked-in syndrome) who retain voluntary eye movement but lack limb motor function [67,69]. In these scenarios, EOG serves as an indirect command input—for example, triggering mode switches or menu selections—rather than providing continuous proportional control of rehabilitation robot joints. In the context of multimodal rehabilitation systems, EOG has appeared primarily as a supplementary modality for intent inference. For instance, Zandigohar et al. [70] incorporated eye-gaze tracking alongside EMG and computer vision in a Bayesian decision-level fusion framework for prosthetic hand control, where gaze direction provided contextual information about the target object to disambiguate grasp intent.

EOG is included in this review for two reasons. First, for patients with severe paralysis who cannot generate sufficient EMG or perform motor imagery tasks reliably, EOG may represent one of the few remaining voluntary signal sources available for human–machine interaction. Second, as multimodal fusion systems increasingly incorporate heterogeneous signal combinations, EOG could serve as an auxiliary channel to supplement primary motor signals (EMG, EEG) in specific clinical scenarios, particularly for high-level task selection or safety override commands. However, its current application in limb motor dysfunction rehabilitation equipment remains limited, and future research is needed to determine whether the inclusion of EOG provides meaningful performance gains in this specific domain.

#### 4.1.4. Motion Capture and Inertial Sensing

In many applications, rehabilitation robots use optical motion capture to measure motion. The technical principle involves affixing optical markers to the essential components of the object for measurement, while simultaneously tracking the motion trajectory of these markers in real time using a multi-angle high-speed camera system. After processing the data, it is possible to get the object’s three-dimensional motion parameters with great accuracy [71]. This technology achieves high-precision quantitative representation of intricate human motion through the combined functionality of optical imaging and spatial positioning algorithms.

The inertial sensing system collects motion parameters by arranging an inertial sensor array at the human joint and calculates the three-dimensional spatial motion attitude parameters of each joint based on the data fusion algorithm. At the end of the 20th century, scientists started to do systematic research on inertial sensing equipment. This technology gradually became a major research focus in the field of motion measurement. The inertial measurement unit (IMU) combines sensors like accelerometers, gyroscopes, and magnetometers to keep track of changing parameters like angular displacement and angular velocity. It also uses algorithms to eliminate noise interference and obtain the quantitative parameters of human joint motion. A representative IMU sensor placement on the lower limb is illustrated in Figure 6d. Finally, it calculates the current motion state and location information of the human body [57].

#### 4.1.5. Electrocardiography (ECG)

Electrocardiography (ECG) records the electrical activity of the heart through surface electrodes, producing a characteristic waveform consisting of the P wave, QRS complex, and T wave [72]. The typical amplitude ranges from 0.5 to 4 mV, with the dominant spectral energy concentrated below 40 Hz [73]. In wearable rehabilitation applications, simplified single-lead or three-lead configurations are commonly adopted for portability. A representative wearable ECG signal acquisition device is shown in Figure 6e.

Unlike EMG and EEG, which directly encode motor intention or muscular activation, ECG provides complementary physiological information about cardiovascular response during rehabilitation training. ECG-derived features such as heart rate (HR) and heart rate variability (HRV) can reflect exercise intensity, fatigue state, and autonomic nervous system activity [74]. In multimodal rehabilitation systems, ECG has been used primarily in two ways: (1) as a safety monitoring signal to detect abnormal cardiac rhythms or excessive exertion during robot-assisted training, and (2) as a supplementary modality fused with motor signals to improve recognition robustness during fatiguing movements. For example, Wang et al. [75] showed that fusing sEMG with ECG improved upper limb motion recognition accuracy from 91.3% to 95.7%, and Yu et al. [76] combined ECG with PPG for stroke risk prediction. However, ECG signals during active rehabilitation are susceptible to motion artifacts, requiring appropriate filtering and denoising techniques [73].

### 4.2. Structure of Multimodal Information Fusion

Multimodal information fusion involves a number of the same or different kinds of sensors and a large amount of data. Due to the existence of a variety of sensors, multimodal information fusion can be used in a variety of ways, and the various ways also have their own characteristics and applicable occasions. According to the different ways of information fusion for multimodal data processing, the structure of multimodal information fusion is mainly divided into centralized, distributed and hybrid forms, which are introduced below.

#### 4.2.1. Centralized Structure

**Figure 7:**
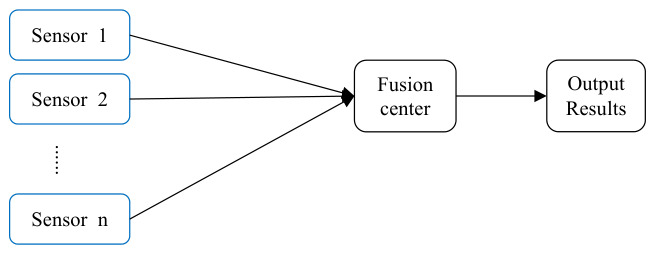
Centralized structure.

In the centralized structure, each sensor does not process its own detected data, but only transmits the detected information to the fusion center, which completes the processing of each step of data fusion. This kind of system has a relatively large communication volume, so it has high requirements for communication ability and is generally suitable for smaller sensor systems; its logical structure is shown in Figure 7.

#### 4.2.2. Distributed Structure

**Figure 8:**
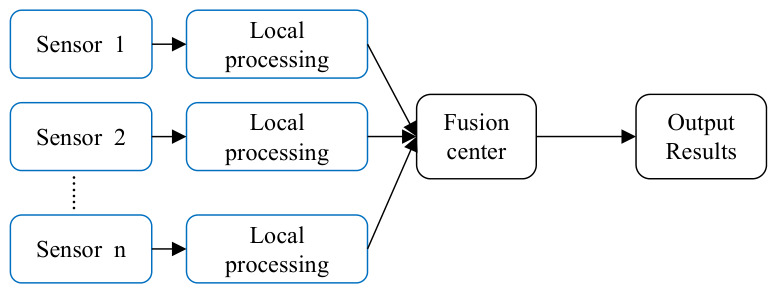
Distributed structure.

In the distributed structure, each sensor completes certain information processing tasks, they only transmit the local processing results to the fusion center, and the fusion center carries out the final fusion processing according to these local results. The system of this structure has a more reasonable distribution of computational tasks and a smaller requirement for the communication channel, but because the fusion center gets the locally processed data, it cannot get the original data, which may result in the loss of part of the information. Its logical structure is shown in Figure 8.

#### 4.2.3. Hybrid Structure

**Figure 9:**
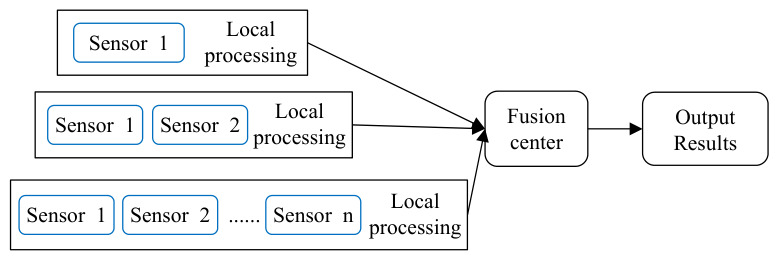
Hybrid structure.

The hybrid multimodal information fusion system structure is shown in Figure 9. This structure combines elements of both the centralized and distributed architectures: some sensors transmit raw data directly to the fusion center, while others perform local processing before forwarding their results. The fusion center then integrates both types of input to produce the final output.

This flexibility is particularly important when a rehabilitation system incorporates both homogeneous and heterogeneous sensor types. For homogeneous sensor groups—such as multi-channel sEMG or multi-axis IMU arrays—the sensors share similar signal characteristics, and their raw data can be directly concatenated and processed jointly at the data or early feature level. For example, Zhang et al. [45] and Meng et al. [54] stacked multi-channel sEMG and accelerometer recordings into a unified matrix for CNN-based processing.

In contrast, heterogeneous sensors—such as EEG paired with sEMG, or EMG paired with vision—differ fundamentally in sampling rate, amplitude scale, and the physiological processes they reflect. Direct data-level fusion is often impractical. Instead, each modality undergoes independent local processing, and only the extracted features or classification outputs are forwarded for integration. The hybrid BMI systems of Kawase et al. [77] and Kiguchi and Hayashi [78] exemplify this approach: EEG and EMG were processed through separate pipelines before their decision outputs were combined. Similarly, Zandigohar et al. [70] processed EMG, vision, and eye-gaze through independent modules before applying Bayesian decision fusion.

The hybrid structure thus balances information preservation and computational efficiency: homogeneous data can be fused at a low level to retain cross-sensor correlations, while heterogeneous data can be fused at a higher level to accommodate signal differences. However, this flexibility increases design complexity, as the grouping of sensors, the fusion level for each group, and the integration strategy must be determined through domain expertise and empirical evaluation.

### 4.3. Common Ways of Fusing Information from Multiple Sources

The fusion method is a crucial component of the multimodal information fusion system. The process of information fusion often has a strong application relevance, and involves more basic theories. Because of these characteristics, no unified theoretical framework for multimodal information fusion has yet been established. In practice, the choice of fusion method is determined by the specific application scenario. So far, researchers in various fields have proposed a series of effective information fusion methods according to the application scenarios they face, and the common ones are listed in Table 1. Each method is described in detail below.

#### 4.3.1. Weighted Average Method

The weighted average method is the simplest and most intuitive information fusion technique. It computes the fused estimate as a weighted linear combination of the outputs from multiple sensors:

(1) $$\hat{x}=\sum_{i=1}^{n}w_{i}\;x_{i},\;\mathrm{subject}\;\mathrm{to}\;\sum_{i=1}^{n}w_{i}=1,\;w_{i}\geq 0,$$

where $x_{i}$ is the measurement from the *i*-th sensor, $w_{i}$ is the corresponding weight, and *n* is the total number of sensors [79]. The weights are typically assigned based on the estimated reliability, signal-to-noise ratio, or inverse variance of each sensor. For example, in a rehabilitation robot system equipped with multiple IMUs, the weighted average can reduce measurement noise by assigning higher weights to sensors with lower noise levels [80]. The main advantages of this method are its computational simplicity and suitability for real-time applications. However, its performance is highly dependent on the accuracy of weight selection, and it assumes a linear relationship among the sensor outputs. In practice, determining the optimal weights often requires prior knowledge of the sensor noise characteristics, which may not always be available or may change over time during rehabilitation sessions [89].

#### 4.3.2. Bayesian Estimation

Bayesian estimation provides a probabilistic framework for information fusion by combining prior knowledge with observed sensor data through Bayes’ theorem [81]:

(2) $$P\left(H|D\right)=\frac{P(D|H)\;P(H)}{P(D)},$$

where P(H∣D) is the posterior probability of hypothesis *H* given observation *D*, P(D∣H) is the likelihood function, P(H) is the prior probability, and P(D) is the marginal likelihood serving as a normalization constant. In the context of multimodal sensor fusion, Bayesian methods recursively update the state estimate as new measurements from different sensors become available. This recursive property makes Bayesian estimation particularly well-suited for sequential decision-making tasks, such as continuous motion intention classification in rehabilitation robots [82]. The Kalman filter, a widely used variant of Bayesian estimation under Gaussian assumptions, has been extensively applied to fuse inertial sensor and force sensor data for state estimation in rehabilitation exoskeletons [89]. The strengths of Bayesian estimation include its rigorous theoretical foundation and its natural ability to quantify uncertainty. However, the method requires accurate prior probability distributions and well-defined likelihood functions, which can be difficult to specify in complex, nonstationary rehabilitation scenarios involving patient-specific physiological variability [82]. Additionally, when the number of hypotheses is large, exact Bayesian inference becomes computationally intractable, necessitating approximate methods such as particle filtering [80].

#### 4.3.3. Dempster–Shafer Evidence Theory

The Dempster–Shafer (D-S) evidence theory, also known as the theory of belief functions, extends Bayesian probability by introducing the concept of a “frame of discernment” and allowing for the assignment of belief masses to subsets of hypotheses rather than only to individual hypotheses [83,84]. For a frame of discernment $\Theta =\{\theta_{1},\theta_{2},\ldots ,\theta_{N}\}$, a basic probability assignment (BPA) function $m:2^{\Theta }\to \left[0,1\right]$ satisfies

(3) $$m\left(\emptyset \right)=0,\;\sum_{A\subseteq \Theta }m\left(A\right)=1.$$

When two independent sources of evidence provide BPAs $m_{1}$ and $m_{2}$, they can be combined using Dempster’s rule of combination:

(4) $$m_{1,2}\left(A\right)=\frac{1}{1-K}\sum_{B\cap C=A}m_{1}\left(B\right)\;m_{2}\left(C\right),\;K=\sum_{B\cap C=\emptyset }m_{1}\left(B\right)\;m_{2}\left(C\right),$$

where *K* is the conflict factor between the two sources [84]. The key advantage of D-S theory over Bayesian estimation is its ability to explicitly represent and distinguish between uncertainty (lack of knowledge) and ignorance (not knowing which hypothesis is correct), through the belief and plausibility functions. This makes D-S theory particularly useful in rehabilitation scenarios where sensor information may be incomplete or partially conflicting, such as when sEMG signals are corrupted by motion artifacts while IMU signals remain reliable [52]. However, D-S theory is computationally expensive, as the power set $2^{\Theta }$ grows exponentially with the number of hypotheses. Furthermore, Dempster’s combination rule may produce counter-intuitive results when the conflict factor *K* is high, which has motivated various modified combination rules in the literature [90,91].

#### 4.3.4. Fuzzy Logic

Fuzzy logic, introduced by Zadeh [85], provides a mathematical framework for reasoning under uncertainty by replacing the binary true/false logic of classical set theory with graded membership functions. In fuzzy logic-based information fusion, sensor measurements are first converted into fuzzy sets through a fuzzification process in which the degree of membership of each measurement to predefined linguistic categories (e.g., “low force,” “medium force,” “high force”) is computed using membership functions [86]. A fuzzy inference engine then applies a set of expert-defined “IF–THEN” rules to the fuzzified inputs and produces fuzzy outputs, which are subsequently defuzzified to yield a crisp decision or control command.

In rehabilitation robotics, fuzzy logic has been widely applied because of its ability to handle the inherent imprecision and variability of physiological signals without requiring a precise mathematical model of the underlying system [46,92]. For example, Chen [92] used fuzzy logic to identify gait phases from fused sEMG and angle sensor data in a lower limb exoskeleton, defining fuzzy rules such as “IF sEMG activation is high AND knee angle is small THEN gait phase is stance.” The primary advantage of fuzzy logic is its interpretability: the rule base can be designed in collaboration with rehabilitation clinicians, making the fusion process transparent and clinically meaningful. However, the design of appropriate membership functions and rule bases requires substantial domain expertise, and the system’s performance is sensitive to the quality of these design choices. Additionally, as the number of input variables and rules increases, the computational complexity and the difficulty of rule optimization grow considerably [86].

#### 4.3.5. Artificial Neural Networks

Artificial neural networks (ANNs) offer a data-driven approach to information fusion that can automatically learn complex, nonlinear mappings between multimodal sensor inputs and desired outputs through training on labeled datasets [87]. A basic feedforward neural network consists of an input layer that receives raw or pre-processed sensor data from multiple modalities, one or more hidden layers that extract and combine features through weighted connections and nonlinear activation functions, and an output layer that produces classification or regression results [88].

In the context of rehabilitation robot control, ANNs have been applied at both the feature level and the decision level. At the feature level, features extracted from different sensor modalities are concatenated into a single input vector and fed into the network for joint classification or regression. For instance, Tang et al. [93] used a backpropagation neural network (BPNN) to fuse sEMG features and end-effector force measurements for joint angle estimation during upper limb rehabilitation. At the decision level, separate networks can be trained for each modality, and their outputs are combined through a meta-classifier or voting scheme [77].

More recent studies have leveraged advanced deep learning architectures, including convolutional neural networks (CNNs) for spatial feature extraction from sensor arrays [44,45], long short-term memory (LSTM) networks for temporal sequence modeling [94], and attention mechanisms for learning dynamic inter-modal relationships [64,66]. These architectures have demonstrated superior performance compared to traditional machine learning methods, particularly in cross-subject generalization and high-dimensional multimodal data processing. However, ANNs require large amounts of labeled training data, which are expensive and time-consuming to collect in clinical rehabilitation settings. They also have high computational requirements, potential overfitting risks with small datasets, and limited interpretability compared to model-based methods such as Bayesian estimation and fuzzy logic [82,88].

In addition to the five methods described above and summarized in Table 1, there are a number of other information fusion approaches that have been employed in related fields, including Kalman filtering [89], generative rules, random set theory, rough set theory, and support vector machine-based methods. These methods each have their own advantages and applicable scenarios, and the choice of fusion method should be determined by the specific requirements of the rehabilitation system, including the types and number of sensors, the computational constraints, the need for real-time performance, and the availability of training data.

## 5. Application of Multimodal Fusion Control in Motor Function Rehabilitation

This section provides a detailed analysis of the 55 representative studies listed in Table 2, organized by their primary signal combinations. This categorization allows for a systematic comparison of how different modality pairings address specific clinical needs in motor function rehabilitation. Within each category, studies are further discussed in terms of their target applications (upper limb, lower limb, or functional assessment), fusion strategies, algorithms, and reported performance where available. A summary of cross-category trends is presented at the end of this section.

### 5.1. EEG and EMG Fusion

The fusion of electroencephalographic (EEG) and electromyographic (EMG) signals represents one of the most physiologically motivated multimodal combinations in rehabilitation robotics. EEG captures cortical motor planning and intention at its neural source, while EMG reflects the peripheral muscular execution of that intention. Their combination therefore spans the full neural–muscular axis of motor control, offering the potential to detect movement intention earlier and more reliably than either signal alone.

#### 5.1.1. Upper Limb Applications

Several studies have explored EEG–EMG fusion for upper limb rehabilitation and assistive control. Sarasola-Sanz et al. [95] proposed a hierarchical hybrid brain–machine interface (BMI) in which EEG-decoded motor intentions were used to trigger and gate EMG-based proportional control of an upper limb rehabilitation robot. This architecture allowed for stroke patients who retained partial muscular function to drive the device with EMG, while EEG served as an enabling signal to ensure that only volitional movements were assisted. The hierarchical design addressed a practical problem: EMG alone may be unreliable in patients with severe paresis, whereas EEG provides a complementary pathway for intention detection.

Kawase et al. [77] adopted a similar hybrid BMI strategy for an upper limb exoskeleton designed for individuals with paresis. In their system, EEG and EMG signals were processed through separate classifiers, and the final control command was determined at the decision level. The system achieved classification accuracies exceeding 80% in online experiments with healthy subjects, demonstrating that the hybrid approach maintained robust control even when EMG signals were weak. Kiguchi and Hayashi [78] extended this paradigm further by integrating sEMG and EEG with joint angle, force/torque, and visual information in a decision-level fusion framework for an upper limb power-assist exoskeleton. Their system used proportional myoelectric control modulated by neural network outputs, illustrating an early attempt at complex, multi-source integration for upper limb assistance.

More recent work has shifted toward deep learning architectures to exploit the complementarity of EEG and EMG more effectively. Shi et al. [64] developed DMEFNet, a dense co-attention mechanism-based multimodal enhanced fusion network, which was applied to a human–exoskeleton interaction interface. By employing a co-attention mechanism, the network learned to weight the contribution of EEG and sEMG features dynamically, achieving classification accuracy of 93.43% for four upper limb movement classes, which outperformed single-modal baselines by 4–7 percentage points. Jiang et al. [66] proposed E^2^FNet, which used multi-scale convolutional modules and cross-attention mechanisms to fuse EEG and EMG features for hand motion intention recognition. On a dataset involving six hand gestures, E^2^FNet achieved an average accuracy of 91.6%, a notable improvement over separate EEG-only (79.8%) and EMG-only (85.3%) classifiers, confirming the benefit of cross-modal fusion at the feature level.

#### 5.1.2. Lower Limb Applications

EEG–EMG fusion has also been investigated for lower limb motion decoding and exoskeleton control. Cui et al. [96] presented a multimodal framework that integrated EEG, EMG, and mechanomyogram (MMG) signals for decoding lower limb motion intentions. Using support vector machine (SVM) classifiers on feature-level fused data, they reported classification accuracies of approximately 92% for distinguishing between walking, stair ascent, and stair descent, compared to 82–86% for any single modality, highlighting the additive value of the three-signal combination.

Hooda et al. [97] fused EEG and sEMG signals for classifying unilateral foot movements relevant to lower limb prosthetic control. They employed a genetic algorithm (GA) for feature selection followed by SVM classification, achieving a classification accuracy of 95.8% with the fused features compared to 88.3% with EEG alone and 91.2% with sEMG alone. Wei et al. [98] investigated gait phase recognition by extracting distinct features from sEMG (time-domain features) and EEG (frequency-band power), fusing them at the feature level, and classifying gait phases using SVM. Their results showed that the bimodal approach improved recognition rates by 3–5% over single-modal methods. Al-Quraishi et al. [65] applied discriminant correlation analysis (DCA) to fuse EEG and EMG feature sets for lower limb movement recognition, followed by linear discriminant analysis (LDA) classification. They reported an accuracy of 98.7% on a four-class movement task, demonstrating that DCA effectively captured the inter-modal correlations between cortical and muscular signals.

Wang et al. [99] presented a more system-oriented contribution, developing a complete control framework for a lower limb exoskeleton rehabilitation robot. EEG signals provided high-level motion mode selection through a BCI module, while EMG signals drove continuous joint torque estimation. The two modalities were integrated at the decision level using a robust adaptive PD controller, and the system was validated in sit-to-stand and walking experiments with healthy subjects.

### 5.2. sEMG and Inertial Sensor Fusion

The combination of surface electromyography (sEMG) with inertial measurement units (IMUs) or accelerometers is the most frequently reported signal pairing in the reviewed literature (11 of 55 studies). This popularity stems from the strong complementarity between the two modalities: sEMG provides information about muscle activation patterns and force generation, while inertial sensors capture the resulting kinematic output, including limb orientation, acceleration, and angular velocity. Together, they offer a more complete picture of the user’s motor state than either modality in isolation.

#### 5.2.1. Gesture and Motion Recognition

A substantial body of work has used sEMG–IMU fusion for gesture and motion recognition, which serves as the upstream task for intention-driven control. Lu et al. [100] developed a wearable gesture recognition prototype using sEMG and a 3-axis accelerometer. Features from both modalities were fused and classified with a scoring-system classifier, achieving 96.7% accuracy across eight hand gestures in a user-dependent setting. Wu et al. [101] extended this approach to American Sign Language recognition, fusing sEMG and IMU (accelerometer and gyroscope) data with SVM classification. Their system recognized 40 sign language words with 96.5% accuracy for user-dependent models and 86.4% for user-independent models, illustrating the challenge of cross-subject generalization.

Wang et al. [102] compared decision tree and random forest classifiers for sign language recognition using the same sEMG–IMU combination, reporting that random forests achieved 95.2% accuracy and were more robust to inter-session variability. Yang et al. [103] proposed an optimized tree-structure classifier for Chinese Sign Language recognition, using decision-level fusion of sEMG and IMU data, and achieved 97.8% recognition accuracy across 121 sign words. Hahne et al. [104] investigated real-time simultaneous control of multiple degrees of freedom in bionic hand prostheses. Using sEMG and IMU with LDA-based feature-level fusion, they demonstrated that the addition of arm posture information from IMU significantly reduced the posture-related degradation of EMG-based control, a finding validated in amputee end users over multi-week usage.

#### 5.2.2. Upper Limb Rehabilitation and Exoskeleton Control

For upper limb rehabilitation specifically, Ren et al. [94] proposed a deep reinforcement learning approach using multi-stream LSTM networks to process IMU and sEMG data for motion prediction in an exoskeleton robot. Their dueling network architecture predicted the next motion state 200 ms ahead with a root mean square error (RMSE) of 3.2° for joint angle estimation. Zhang et al. [105] fused sEMG and IMU data using hand-crafted feature engineering and regression models for quantitative assessment of upper limb muscle spasticity in post-stroke patients. Their approach achieved a Pearson correlation of 0.89 with clinical Modified Ashworth Scale scores, providing an objective and continuous spasticity metric. Zhang et al. [45] developed the Multimodal Fusion Convolutional Neural Network (MFCNN), which processed sEMG and accelerometer signals through parallel convolutional streams before concatenation and classification. In a cross-subject upper limb motion classification task involving eight movement types, MFCNN achieved 87.0% accuracy, outperforming both single-modal CNN baselines and conventional feature-engineering approaches. Song et al. [106] combined IMU, sEMG, and force myography (FMG) in a wearable multimodal system for hand movement training after stroke. The three-modality fusion enabled real-time tracking of both finger kinematics and grip force, which was integrated into a serious games rehabilitation platform for patient engagement.

#### 5.2.3. Lower Limb Applications

The sEMG–IMU combination has also been applied to lower limb rehabilitation. Cheng et al. [107] fused sEMG with acceleration signals for activity monitoring and fall detection using LDA with a Gaussian kernel to classify daily activities with 92% accuracy. Meng et al. [54] systematically investigated the role of sEMG in enhancing IMU-based locomotion mode recognition for the lower limb. By fusing time-domain sEMG features with IMU kinematic features and applying LDA classification, they demonstrated that the addition of sEMG improved the recognition accuracy of locomotion modes (level walking, stair ascent/descent, ramp ascent/descent) by 4.3% compared to IMU alone, reaching 96.1% overall accuracy.

#### 5.2.4. Functional Assessment

Chen et al. [108] applied sEMG–IMU fusion specifically to clinical assessment, developing a method for quantifying upper limb spasticity during voluntary movement. They extracted biomechanical features from IMU data and neuromuscular features from sEMG, fused them at the feature level, and used support vector regression (SVR) to predict clinical spasticity scores. The system achieved a mean absolute error of 0.35 on the Modified Ashworth Scale, suggesting its potential as an objective clinical assessment tool.

### 5.3. sEMG Combined with Force, Torque, or Joint Angle Sensors

A third major category of multimodal studies pairs sEMG with mechanical sensors that directly measure the forces and joint kinematics involved in movement. Unlike inertial sensors, which infer kinematics from acceleration and angular velocity, force sensors and encoders provide direct measurements of interaction forces and joint positions, making this combination particularly suited for closed-loop control of rehabilitation robots.

#### 5.3.1. Prosthetic and Lower Limb Exoskeleton Control

Huang et al. [109] presented one of the earliest and most influential studies in this category, fusing EMG signals with ground reaction force (GRF) data for continuous locomotion-mode identification in prosthetic legs. Using SVM, they classified seven locomotion modes (level walking, stair ascent/descent, ramp ascent/descent, sitting, standing) with 96.4% overall accuracy during transitions, demonstrating that the neuromuscular–mechanical fusion captured mode transitions more reliably than either signal alone. Fan and Yin [110] combined sEMG with equilibrium-point-based position (EPP) force-position signals for active and progressive exoskeleton rehabilitation of the lower limb, using an active-compliance control strategy that adapted the robot’s assistance level based on the patient’s muscular effort. Chen [92] fused sEMG with angle sensors in a lower limb exoskeleton, using fuzzy logic to identify gait phases and generate appropriate assistance torques.

#### 5.3.2. Upper Limb Rehabilitation and Assessment

Tang et al. [93] fused sEMG with end-effector force measurements using backpropagation neural networks (BPNNs) for joint angle estimation under load variation during upper limb rehabilitation. Their model reduced the estimation error caused by external loads by 38% compared to sEMG-only estimation. Yeh et al. [111] developed a quantitative spasticity assessment method for the knee joint by fusing sEMG with angle sensor data through phase–amplitude coupling (PAC) analysis, providing a neurophysiologically grounded metric for spasticity severity. Hu [112] applied Hilbert–Huang Transform (HHT) and marginal spectrum entropy analysis to fused sEMG and angle sensor data for portable spasticity measurement in upper limb exoskeleton users, achieving a correlation coefficient of 0.91 with clinical assessment scores.

#### 5.3.3. Hand Function Rehabilitation

Ju et al. [113] proposed an empirical copula function for decision-level fusion of sEMG, finger tracking angle, and contact force to recognize hand manipulation intent. The copula-based fusion captured the nonlinear dependencies between the three modalities and achieved 95.5% recognition accuracy across six grasp types. Yang et al. [114] analyzed muscle synergy patterns from fused sEMG and force sensor data during sit-to-stand movements, extracting temporal features that reflected the motor impairment level of post-stroke patients. Their analysis revealed that the temporal activation profiles of muscle synergies were significantly altered in patients compared to healthy controls, providing a quantitative basis for impairment characterization.

Wang et al. [115] developed a multi-layer assessment system for upper limb spasticity that integrated sEMG, IMU, and force sensor data. The system extracted features at multiple levels (muscle activation, joint kinematics, and interaction force) and used them to generate a comprehensive spasticity profile. Xu et al. [116] addressed compensatory motion detection during rehabilitation by fusing sEMG, force, and angular displacement data with SVM classification, achieving 94.6% detection accuracy. Duanmu et al. [117] combined sEMG with force sensors in a perceptual feedback system for hand function rehabilitation, using machine learning to map the fused sensory data to appropriate haptic feedback for the patient.

### 5.4. Kinematic and Mechanical Sensor Fusion

A number of studies have employed multimodal fusion based entirely on kinematic and mechanical sensors without bioelectrical signals. These systems typically combine IMUs, joint encoders, force-sensitive resistors (FSRs), or pressure sensors. While they do not capture the neural or muscular origins of movement, they offer practical advantages in terms of signal reliability, ease of deployment, and robustness against the artifacts and variability that often affect bioelectrical recordings.

#### 5.4.1. Lower Limb Gait Analysis and Exoskeleton Control

Wang [118] fused force sensors, angle sensors, and IMU data for sensor-guided gait synchronization in a weight-support lower extremity exoskeleton, using adaptive oscillators to synchronize robot assistance with the user’s natural gait rhythm. Lee et al. [44] combined joint encoder, IMU, and FSR data in a CNN-based system for real-time slope prediction in a robotic knee exoskeleton. The CNN processed time-series segments from the fused sensor data and predicted upcoming terrain slope with a mean absolute error of 1.8°, enabling anticipatory adjustment of exoskeleton assistance. Notably, the model generalized across users without subject-specific calibration.

Wei et al. [119] designed a synergy-based control system for a lower limb exoskeleton that fused GRF, IMU, and EMG signals. They used impedance matching derived from muscle synergy patterns to modulate the exoskeleton’s joint impedance in real time, transferring motor skills from healthy subjects to the assistive device. Gavrilovic and Jankovic [120] fused IMU and force sensor data from instrumented insoles to detect temporal synergies in gait cyclograms using PCA, providing a compact representation of gait variability that could serve as a monitoring tool for rehabilitation progress. Li et al. [121] used a body sensor network comprising IMUs and kinematic sensors to quantify the effect of rehabilitation therapy on lower limb motor function in children with spastic diplegia, demonstrating measurable improvements in gait symmetry and stride regularity after intervention. Zhao et al. [122] combined flexible sEMG sensors and piezoresistive sensing devices (PSDs) in a wearable insole for Internet-of-Things (IoT)-based locomotion mode recognition, using LDA to classify five locomotion modes with 93.2% accuracy. Huang et al. [123] integrated motor encoder and EMG data for individualized sit-to-stand assistance in a lower extremity exoskeleton, using root mean square (RMS) analysis of EMG to adapt the assistance trajectory to each user’s effort level.

#### 5.4.2. Upper Limb and Hand Applications

Wojewoda et al. [124] employed data-level fusion of IMU and optical motion capture data for position tracking of a passive upper limb rehabilitation robot, using the high-accuracy optical system as a reference to validate and calibrate the portable IMU system. Yu [125] developed a wearable sensor network with accelerometers and flex sensors for remote quantitative assessment using the Fugl-Meyer scale, employing extreme learning machine (ELM) regression to map sensor features to clinical scores with a Pearson correlation of 0.92. Yu et al. [126] fused Leap Motion (optical) and pressure sensor data for bilateral upper limb rehabilitation, enabling simultaneous tracking of hand posture and grip force in bimanual coordination training. Bobin et al. [127] integrated IMU and FSR data in a smart cup device for monitoring stroke patient activities during daily life, using machine learning to classify functional hand activities with 89.5% accuracy.

#### 5.4.3. Functional Assessment

Yalçın et al. [128] developed a spasticity assessment glove that fused IMU, encoder, and pressure sensor data, using supervised machine learning to mitigate motion artifacts and provide clinician-grade spasticity measurement in a wearable form factor. Miao et al. [129] proposed an upper limb functionality assessment system that fused IMU and camera data, combining dynamic time warping–*k*-nearest neighbor (DTW-KNN) for posture matching and LSTM for temporal pattern recognition. Their dual-method approach achieved an assessment accuracy within 0.5 points of expert clinician ratings on the Fugl-Meyer scale.

### 5.5. Vision-Augmented and Other Emerging Multimodal Combinations

Several studies have explored less conventional signal combinations, incorporating visual information, physiological signals beyond EMG and EEG, or highly heterogeneous sensor arrays. These approaches address specialized application scenarios where standard signal pairs may be insufficient.

#### 5.5.1. Vision-Integrated Systems

Hu et al. [130] developed a sensory–motor fusion system for robotic hand-eye grasping that integrated sEMG, hybrid force sensing, and visual information at the decision level through hierarchical control. The visual module identified objects and planned grasp configurations, sEMG decoded the operator’s intended grasp type, and force sensing provided closed-loop grasp stability control. Zandigohar et al. [70] extended vision-based multimodal fusion to prosthetic hand control by combining EMG, computer vision, and eye-gaze tracking. Using Bayesian evidence fusion at the decision level, the system integrated bottom-up grasp affordance information from vision with top-down intention signals from EMG and gaze, achieving 89.5% grasp success rate in activities-of-daily-living tasks, a significant improvement over EMG-only control (72.3%).

Cui et al. [131] fused sEMG, force sensors, and camera-based kinematic data for simultaneous recognition and assessment of post-stroke hemiparetic gait, achieving 93.7% gait pattern recognition accuracy and demonstrating strong correlation between computed gait indices and clinical assessment scores. Park et al. [132] proposed mFAST, a multimodal automatic stroke evaluation system that fused video, audio, and inertial sensor data using machine learning, targeting rapid pre-hospital stroke screening in time-critical treatment scenarios.

#### 5.5.2. Novel Biosignal Combinations

Guo et al. [133] investigated the combination of sEMG and near-infrared spectroscopy (NIRS) for upper limb prosthetic control. NIRS measures hemodynamic changes in muscle tissue that correlate with sustained force production. Using LDA and SVM classifiers on the fused features, they reported that the bimodal system improved hand gesture classification accuracy by 5.6% over sEMG alone, with particular benefits for distinguishing between gestures requiring similar muscle activation patterns but different force levels. Wang et al. [75] fused sEMG and electrocardiogram (ECG) signals for upper limb motion pattern recognition using an Inception-Sim CNN architecture. The inclusion of ECG, which reflects cardiovascular response to physical exertion, improved recognition accuracy from 91.3% (sEMG only) to 95.7%, especially during sustained or fatiguing movements where EMG signal quality degrades. Yu et al. [76] combined ECG and photoplethysmography (PPG) signals using a CNN-LSTM architecture for stroke risk prediction, achieving a classification accuracy of 96.4% in distinguishing between stroke-prone and healthy cardiovascular profiles.

#### 5.5.3. Other Specialized Systems

Park et al. [134] developed a patient-driven robotic hand rehabilitation device that fused pressure sensor and infrared (IR) sensor data at the decision level using threshold-based triggering. The system detected the patient’s residual grasp attempt through pressure sensing and finger proximity through IR sensing, triggering robotic assistance only when both signals indicated volitional effort. Park et al. [135] designed a robotic hand orthosis with shared autonomy, fusing sEMG, IMU, and FSR data at the decision level to balance user control with robotic compensation. The shared autonomy framework allowed for the system to interpolate between full user control and full robotic assistance based on the estimated severity of the user’s impairment, validated with chronic stroke patients.

### 5.6. Summary of Cross-Category Trends

Across the 55 studies reviewed, several consistent trends and comparative observations emerge regarding signal combinations, fusion strategies, algorithms, and application targets.

Signal combination preferences: sEMG-based combinations dominate the literature, appearing in over 70% of all reviewed studies. The most common pairings are sEMG + IMU (11 studies) and EEG + EMG (10 studies), reflecting their strong physiological complementarity. sEMG + force/mechanical sensor combinations constitute the third largest group (nine studies). Vision-augmented systems remain relatively rare (four studies), but show a growing trend in recent years.

Fusion level distribution: Feature-level fusion is by far the dominant strategy, employed in approximately 78% of the reviewed studies (43 of 55). Decision-level fusion accounts for approximately 20% (11 studies), predominantly in systems that combine heterogeneous or semantically distinct modalities (e.g., EEG and EMG processed by separate classifiers, or EMG with vision). Data-level fusion appears in only one study [124], consistent with the high computational cost and strict sensor synchronization requirements of this approach.

Algorithm evolution: The reviewed studies exhibit a clear temporal progression from traditional machine learning to deep learning methods. Studies published before 2018 predominantly used LDA, SVM, decision trees, fuzzy logic, or threshold-based methods. From 2019 onward, CNN, LSTM, and attention-based architectures (DMEFNet, E^2^FNet, Inception-Sim) have become increasingly common. Deep learning methods generally report higher classification accuracies (often exceeding 90%) and show better generalization in cross-subject scenarios, although they require larger datasets and more computational resources.

Upper vs. lower limb: Upper limb applications (approximately 28 studies) slightly outnumber lower limb applications (approximately 20 studies), with the remainder targeting whole-body assessment or specialized tasks. Upper limb studies tend to prioritize fine motor control and intention recognition (e.g., hand gesture, grasp type), while lower limb studies focus on gait phase recognition, locomotion mode classification, and terrain adaptation.

Clinical validation gap: Despite the generally strong technical performance reported across all categories, the majority of studies were validated exclusively with healthy subjects in laboratory settings. Only a minority [95,104,105,106,114,131,135] included patient populations (primarily chronic stroke survivors) in their experimental protocols. This gap between technical demonstration and clinical validation remains the most significant barrier to translation.

**Table 2:** Representative devices for multimodal fusion control in motor function rehabilitation in recent years.

No.	Reference	Year	Application	Multimodal Signals	Fusion Level	Methods
1	Huang et al. [109]	2011	Prosthetic leg control	EMG, GRF	Feature-level	SVM
2	Cheng et al. [107]	2012	Lower limb rehab.	sEMG, acceleration	Feature-level	LDA (Gaussian kernel)
3	Kiguchi et al. [78]	2012	Upper limb exoskeleton	sEMG, EEG, angle, force/torque, vision	Decision-level	Proportional myoelectric, NN
4	Fan et al. [110]	2013	Lower limb exo. rehab.	sEMG, force-position (EPP)	Feature-level	Active-compliance control
5	Park, W. [134]	2013	Robotic hand rehab.	Pressure sensor, IR sensor	Decision-level	Threshold-based triggering
6	Lu et al. [100]	2014	Gesture recognition	sEMG, IMU (3-axis accel.)	Feature-level	Scoring-system classifier
7	Ju et al. [113]	2014	Hand intent recognition	sEMG, track angle, contact force	Decision-level	Empirical copula function
8	Chen, D. [92]	2014	Lower limb exoskeleton	sEMG, angle sensors	Feature-level	Fuzzy logic (gait phase)
9	Tang et al. [93]	2016	Upper limb rehab.	sEMG, end force	Feature-level	BPNN
10	Wu et al. [101]	2016	Gesture recognition	sEMG, IMU (accel., gyro.)	Feature-level	SVM
11	Wang et al. [102]	2016	Gesture recognition	sEMG, IMU (accel., gyro.)	Feature-level	Decision tree and random forest
12	Yu, L. [125]	2016	Upper limb/gloves	Accelerometer, flex sensors	Feature-level	ELM regression
13	Wang, D. [118]	2016	Lower limb exoskeleton	Force, angle sensor, IMU	Feature-level	Adaptive oscillators
14	Yu, N. [126]	2016	Upper limb	Leap Motion, pressure sensor	Feature-level	Bilateral training
15	Yeh et al. [111]	2016	Knee	sEMG, angle sensor	Feature-level	PAC analysis
16	Wojewoda et al. [124]	2016	Upper limb rehab. robot	IMUs, optical motion capture	Data-level	Sensor fusion and validation
17	Guo et al. [133]	2017	Upper limb prosthetic	sEMG, NIRS	Feature-level	LDA, SVM
18	Yang et al. [103]	2017	Gesture recognition	sEMG, IMU (accel., gyro.)	Decision-level	Optimized tree-structure
19	Hu et al. [130]	2017	Robotic hand-eye grasp	sEMG, hybrid force, vision	Decision-level	Hierarchical control
20	Cui et al. [96]	2017	Lower limb decoding	EEG, EMG, MMG	Feature-level	SVM
21	Sarasola-Sanz et al. [95]	2017	Upper limb motor rehab.	EEG, EMG	Feature-level	Hierarchical control
22	Kawase et al. [77]	2017	Upper limb exoskeleton	EEG, EMG	Decision-level	Hybrid BMI
23	Hahne et al. [104]	2018	Real-time gesture recog.	sEMG, IMU	Feature-level	LDA
24	Hu, B. [112]	2018	Upper limb exoskeleton	sEMG, angle sensor	Feature-level	HHT and entropy analysis
25	Cui, C. [131]	2018	Lower limb/floor	sEMG, force sensor, camera	Feature-level	ML classification and regression
26	Bobin et al. [127]	2018	Hand	IMU, FSRs	Feature-level	ML activity recognition
27	Ren et al. [94]	2019	Upper limb rehab. exo.	IMU, sEMG	Feature-level	Multi-stream LSTM dueling
28	Wei et al. [119]	2019	Lower limb exoskeleton	GRF, IMU, EMG	Feature-level	Impedance matching
29	Yang, N. [114]	2019	Upper and lower limb	sEMG, force sensor	Feature-level	Muscle synergy analysis
30	Zhang, X. [105]	2019	Upper limb	sEMG, IMU	Feature-level	Feature eng. and regression
31	Park, S. [135]	2019	Hand	sEMG, IMU, FSRs	Decision-level	Shared autonomy
32	Hooda et al. [97]	2020	Lower limb prosthetics	EEG, sEMG	Feature-level	GA and SVM
33	Wei et al. [98]	2020	Gait phase recognition	sEMG, EEG	Feature-level	SVM
34	Li, J. [121]	2020	Lower limb	IMU, kinematic data	Feature-level	Quantitative gait analysis
35	Park, E. [132]	2020	Upper and lower limbs, face	Video, audio, inertial sensor	Feature-level	Multimodal ML assessment
36	Lee et al. [44]	2021	Knee exoskeleton	Encoder, IMU, FSR	Feature-level	CNN
37	Huang et al. [123]	2021	Lower exo. (sit-to-stand)	Motor encoder, EMG	Feature-level	RMS analysis
38	Al-Quraishi et al. [65]	2021	Lower limb movement	EEG, EMG	Feature-level	DCA, LDA
39	Wang, C. [115]	2021	Upper limb assessment	sEMG, IMU, force sensor	Feature-level	Multi-layer assessment
40	Yalçın et al. [128]	2021	Spasticity assess. glove	IMU, encoder, pressure sensor	Feature-level	Supervised ML
41	Zhao et al. [122]	2021	Lower limb/insole	sEMG, PSD	Feature-level	Flexible sensors and LDA
42	Meng et al. [54]	2021	Lower limb	sEMG, IMU	Feature-level	Feature extraction and LDA
43	Chen, Y. [108]	2021	Spasticity assessment	sEMG, IMU	Feature-level	Feature extraction and SVR
44	Miao et al. [129]	2021	Upper limb assessment	IMU, camera	Feature-level	DTW-KNN and LSTM
45	Shi et al. [64]	2022	Rehab. training interface	EEG, sEMG	Feature-level	DMEFNet
46	Gavrilovic et al. [120]	2022	Lower limb /insole	IMU, force sensor	Feature-level	Cyclogram and PCA
47	Song et al. [106]	2022	Hand rehab. training	IMU, sEMG, FMG	Feature-level	Serious games
48	Xu et al. [116]	2022	Seat	sEMG, force, angular disp.	Feature-level	SVM
49	Yu, J. [76]	2022	Chest/finger	ECG, PPG	Feature-level	CNN and LSTM
50	Wang et al. [75]	2023	Upper limb pattern recog.	sEMG, ECG	Feature-level	Inception-Sim (CNN)
51	Zhang et al. [45]	2023	Upper limb classification	sEMG, accelerometer	Feature-level	MFCNN
52	Zandigohar et al. [70]	2024	Prosthetic hand control	EMG, vision, eye-gaze	Decision-level	Bayesian evidence fusion
53	Duanmu et al. [117]	2024	Hand function rehab.	sEMG, force sensors	Feature-level	ML, perceptual feedback
54	Jiang et al. [66]	2024	Hand motion intent recog.	EEG, EMG	Feature-level	E^2^FNet (CNN + cross-attn.)
55	Wang et al. [99]	2025	Lower limb exo. robot	EEG, EMG	Decision-level	Robust adaptive PD control

## 6. Discussion

The use of multimodal information fusion technology in the control systems of motor dysfunction rehabilitation devices is methodically described in this review. It is clear from the literature that integrating multi-sensor data is a basic technical approach to enhancing the flexibility and interactivity of rehabilitation systems. However, a more thorough examination reveals that there are significant trade-offs between various fusion strategies, that fusion algorithms are undoubtedly improving, and that there are still issues that make this technology difficult to apply in clinical settings. The following discussion addresses the key technical limitations, unresolved challenges, and possible future directions identified through the analysis of the 55 reviewed studies.

### 6.1. Trade-Offs Among Fusion Levels

The widespread use of feature-level fusion is a noteworthy trend in the reviewed literature, employed in approximately 78% of the studies (Table 2). This approach appears to be a workable compromise between satisfying the requirements of real-time processing and preserving the richness of raw data. Due to the large amount of raw data that must be processed, data-level fusion is computationally demanding; indeed, only one study [124] adopted data-level fusion, relying on a controlled laboratory environment with optical motion capture as a reference. In contrast, feature-level fusion aggregates key features from every stream of sensor data. For wearable and portable rehabilitation systems, this reduces the quantity of data points and processing power required, which is crucial.

However, the effectiveness of feature-level fusion depends heavily on the quality of the extracted features. As observed in studies such as [54,105], hand-crafted time-domain and frequency-domain features can capture known physiological patterns, but they may miss subtle cross-modal correlations that are not anticipated by the designer. Decision-level fusion, adopted in 11 studies, is the most fault-tolerant and computationally efficient, but it also runs the risk of losing detailed inter-modal information. For example, the hybrid BMI systems of [77,78] processed EEG and EMG through entirely separate classification pipelines, which simplified the system architecture but precluded the discovery of joint EEG–EMG temporal patterns that may carry additional discriminative information, as later demonstrated by the feature-level attention models of [64,66].

A practical consequence of this trade-off is that no single fusion level is universally optimal. Systems targeting real-time wearable control with limited computational resources may favor decision-level fusion for its modularity and low latency, whereas offline assessment systems that prioritize diagnostic accuracy may benefit from data-level or deep feature-level fusion. Future work should consider adaptive or hierarchical fusion architectures that dynamically adjust the fusion depth based on the computational budget and the reliability of each sensor stream at a given moment.

### 6.2. Algorithm Limitations and the Cost of Deep Learning

The reviewed studies exhibit a clear temporal progression from traditional machine learning to deep learning methods. Studies published before 2018 predominantly used LDA, SVM, decision trees, fuzzy logic, or threshold-based methods. From 2019 onward, CNN, LSTM, and attention-based architectures (DMEFNet [64], E^2^FNet [66], Inception-Sim [75]) have become increasingly common. Deep learning methods generally report higher classification accuracies (often exceeding 90%) and show better generalization in cross-subject scenarios, though they require larger datasets and more computational resources.

However, the adoption of deep learning in rehabilitation robotics introduces several underappreciated problems. First, the labeled training data required by these models are expensive and time-consuming to collect in clinical settings. Unlike computer vision or natural language processing, where large public datasets are available, rehabilitation data involve patient-specific physiological recordings that are difficult to standardize and share across institutions due to ethical and privacy constraints. Most of the reviewed deep learning studies [45,64,66,94] were trained and validated on datasets collected from fewer than 20 subjects, raising concerns about overfitting and limited statistical power.

Second, the computational demands of deep networks conflict with the real-time and low-power requirements of wearable rehabilitation devices. Although model compression techniques such as pruning and quantization exist, their application to multimodal rehabilitation models has received little attention in the reviewed literature.

Third, deep learning models are often treated as black boxes, making it difficult for clinicians to understand why a particular movement intention was classified in a certain way. This lack of interpretability is a significant barrier to clinical trust and regulatory approval. Traditional methods such as fuzzy logic [92] offer inherently interpretable rule bases that can be designed in collaboration with clinicians, an advantage that should not be discarded in favor of marginal accuracy gains.

### 6.3. Signal Quality and Robustness in Real-World Conditions

A recurring limitation across nearly all reviewed studies is the assumption of relatively clean, laboratory-grade signal acquisition. In practice, sEMG signals are highly susceptible to electrode shift, skin impedance changes, sweat accumulation, and crosstalk from adjacent muscles [104]. EEG signals suffer from even greater vulnerability to motion artifacts, ocular contamination, and environmental electromagnetic interference [64,65]. ECG signals during active movement are similarly degraded by motion artifacts [73,75].

These signal quality issues have direct consequences for fusion system performance. For instance, Zhang et al. [105] reported that sEMG–IMU fusion accuracy for spasticity assessment degraded by approximately 8% when electrode placement deviated by more than 1 cm from the optimal position. Hahne et al. [104] found that the benefit of adding IMU data to sEMG was most pronounced precisely when sEMG quality was compromised by arm posture changes, suggesting that multimodal fusion can partially compensate for single-modal degradation, but only if the complementary modality remains reliable. When multiple modalities degrade simultaneously, as may occur during vigorous rehabilitation exercises, no reviewed system has demonstrated robust performance.

Potential solutions include adaptive signal quality monitoring that dynamically adjusts fusion weights based on real-time signal-to-noise ratio estimates, as well as the development of artifact-robust feature extraction methods. The spasticity assessment glove of Yalçın et al. [128], which explicitly incorporated artifact mitigation into its sensor fusion pipeline, represents a promising direction, although its approach has not yet been generalized to other applications.

### 6.4. Cross-Subject Generalization

The majority of reviewed studies reported user-dependent performance, where models were trained and tested on data from the same individual. When cross-subject evaluation was conducted, performance consistently dropped. For example, Wu et al. [101] observed a decrease from 96.5% to 86.4% accuracy in sign language recognition when switching from user-dependent to user-independent models. Zhang et al. [45] reported 87.0% cross-subject accuracy for upper limb motion classification, which, while competitive, still falls short of the user-dependent accuracies (often >95%) reported by simpler systems.

This generalization gap arises from substantial inter-individual variability in muscle anatomy, subcutaneous tissue thickness, neural activation patterns, and movement strategies, all of which are further amplified in patient populations with heterogeneous impairment profiles. Transfer learning and domain adaptation techniques, which have proven to be effective in other biosignal processing domains, have been largely unexplored in the multimodal rehabilitation fusion context. Calibration procedures that require only a few minutes of user-specific data to fine-tune a pre-trained model could offer a practical compromise between full user independence and lengthy subject-specific training.

### 6.5. Clinical Validation Gap

Perhaps the most critical shortcoming identified in this review is the disconnect between technical demonstration and clinical validation. Despite the generally strong classification accuracies reported across all categories, the majority of studies were validated exclusively with healthy subjects in laboratory settings. Only a minority [95,104,105,106,114,131,135] included patient populations in their experimental protocols, and even these studies typically involved small samples of chronic stroke patients (often fewer than ten) performing constrained tasks.

This gap is problematic for several reasons. First, the motor patterns of patients with neurological impairments differ qualitatively—not just quantitatively—from those of healthy subjects. Patients may exhibit spasticity, synkinesis, compensatory movements, and highly variable muscle activation patterns that are not represented in healthy-subject training data. Second, the practical usability of multimodal sensor systems in clinical or home environments has been inadequately assessed. Issues such as sensor donning/doffing time, patient comfort during prolonged use, and the need for technical supervision remain largely unaddressed. Third, the clinical meaningfulness of classification accuracy as a performance metric is questionable: a system that achieves 95% gesture recognition accuracy may still be clinically unusable if its 5% error rate produces unsafe or frustrating robot behaviors during actual rehabilitation sessions.

Addressing this gap requires multi-site clinical trials with adequately powered patient cohorts, longitudinal studies that track rehabilitation outcomes over weeks or months rather than single-session experiments, and the development of clinically relevant performance metrics that go beyond classification accuracy to include measures such as task completion time, patient effort, safety events, and functional improvement scores.

### 6.6. Sensor Wearability and System Integration

From a practical deployment perspective, many of the reviewed systems require multiple sensor types to be attached to the patient’s body, each with its own wiring, power supply, and data acquisition unit. For example, sEMG–IMU fusion systems typically require adhesive electrode patches, IMU modules strapped to limb segments, and a central data acquisition box, collectively creating a setup that may take 15–30 min to prepare and is uncomfortable for extended use. EEG-based systems add further complexity through the need for conductive gel, scalp preparation, and a multi-channel EEG cap.

This hardware burden directly limits clinical adoption. Rehabilitation sessions typically last 30–60 min, and if a significant fraction of that time is consumed by sensor setup, the net training time is substantially reduced. Recent advances in flexible and textile-integrated sensors, such as the flexible sEMG and PSD insole system of Zhao et al. [122], offer a potential path toward less obtrusive sensor integration. Similarly, dry-electrode EEG systems and behind-the-ear EEG devices could reduce setup time for brain signal acquisition, although their signal quality remains inferior to conventional wet-electrode systems. Future multimodal systems should prioritize minimizing the number of discrete sensor units and exploring integrated sensor platforms that combine multiple sensing modalities in a single wearable form factor.

### 6.7. Future Directions

Based on the above analysis, several concrete research directions can be identified:

1. Adaptive fusion frameworks: Rather than committing to a fixed fusion level, future systems could implement hierarchical architectures that automatically select the appropriate fusion depth (data, feature, or decision level) based on real-time assessment of signal quality, computational load, and task demands.
2. Lightweight and interpretable models: The development of compact neural network architectures optimized for edge deployment on wearable processors, combined with explainability techniques (e.g., attention visualization, rule extraction) that make model decisions transparent to clinicians, is essential for clinical acceptance.
3. Transfer learning and few-shot calibration: Pre-trained multimodal models that can be rapidly adapted to new users with minimal calibration data would address the cross-subject generalization problem without imposing excessive setup burden on patients.
4. Standardized benchmarks and open datasets: The field currently lacks standardized multimodal datasets with patient populations. Establishing such benchmarks would enable fair comparison across methods and accelerate progress, analogous to the role of ImageNet in computer vision.
5. Longitudinal clinical validation: Moving beyond single-session laboratory experiments to multi-week or multi-month clinical trials with patient populations is not only necessary to evaluate technical performance, but also therapeutic efficacy, user acceptance, and long-term system reliability.
6. Integrated wearable platforms: Reducing the sensor hardware burden through multi-modal integrated sensor modules—combining, for example, sEMG, IMU, and force sensing in a single flexible patch—would improve clinical usability and patient compliance.

## 7. Conclusions

Summarizing the results of the above literature review, multimodal information fusion has important application value in rehabilitation equipment designed for people with motor dysfunction. By integrating information from multiple sensors such as brain, muscles, eyes, inertial sensors, pressure sensing, and acceleration sensors, the rehabilitation equipment can be more intelligent and personalized to assist the patients in their rehabilitation training and improve the rehabilitation effect and quality of life. However, current research still faces some challenges, such as the complexity and feasibility of the technology, the optimization of data fusion algorithms, and the promotion of clinical practice. Future efforts should focus on advancing the research and development of multimodal information fusion technology to promote its wide application in the field of motor dysfunction rehabilitation. At the same time, researchers should work closely with clinicians and patients to gain a deeper understanding of their needs and feedback so as to ensure that the functions and performances of rehabilitation devices can better meet the needs of practical applications. Through continuous cooperation and innovation, we expect to promote the further development of multimodal information fusion in the field of rehabilitation equipment and provide patients with more effective, convenient, and personalized rehabilitation services.

## Figures and Tables

**Table 1:** Common ways of fusing information from multiple sources.

Fusion Methodology	Characteristic
Weighted Average Method [79,80]	The method is relatively simple, better in real time and suitable for dynamic environments. Required weighting factors are not easy to obtain.
Bayesian Estimation [81,82]	Expresses uncertainty directly as probability, suitable for static environments. Requires a priori probabilities and mutually exclusive decision results.
Dempster–Shafer Theory [83,84]	Can distinguish between uncertain and unknown information, and has a high degree of fault tolerance. Highly computationally intensive, requiring that the combined evidence be independent of each other.
Fuzzy Logic [85,86]	Uncertainty can be directly expressed in the reasoning process, strong regularity. No need to build precise mathematical models.
Artificial Neural Networks [87,88]	Parallel processing capability and fault tolerance. Requires a large number of learning samples, high computational effort.

## Data Availability

No new data were created or analyzed in this study. Data sharing is not applicable to this article.

## Abbreviations

The following abbreviations are used in this manuscript:

BCI	Brain–Computer Interface
BMI	Brain–Machine Interface
BPNN	Backpropagation Neural Network
CNN	Convolutional Neural Network
DALYs	Disability-Adjusted Life Years
DCA	Discriminant Correlation Analysis
DMEFNet	Dense Co-attention Mechanism-based Multimodal Enhanced Fusion Network
DTW	Dynamic Time Warping
ECG	Electrocardiogram
EEG	Electroencephalogram
ELM	Extreme Learning Machine
EMG	Electromyogram
EOG	Electrooculogram
EPP	Equilibrium-Point-based Position
FMG	Force Myography
FSR	Force-Sensitive Resistor
GA	Genetic Algorithm
GRF	Ground Reaction Force
HHT	Hilbert–Huang Transform
ICA	Independent Component Analysis
IMU	Inertial Measurement Unit
IR	Infrared
KNN	K-Nearest Neighbor
LDA	Linear Discriminant Analysis
LSTM	Long Short-Term Memory
MFCNN	Multimodal Fusion Convolutional Neural Network
ML	Machine Learning
MMG	Mechanomyogram
MUAP	Motor Unit Action Potential
NEMG	Needle Electromyogram
NIRS	Near-Infrared Spectroscopy
NN	Neural Network
PAC	Phase–Amplitude Coupling
PCA	Principal Component Analysis
PPG	Photoplethysmography
PSD	Piezoresistive Sensing Device
RMS	Root Mean Square
sEMG	Surface Electromyogram
SVM	Support Vector Machine
SVR	Support Vector Regression
